# Binding of Hanatoxin to the Voltage Sensor of Kv2.1

**DOI:** 10.3390/toxins4121552

**Published:** 2012-12-18

**Authors:** Rong Chen, Anna Robinson, Shin-Ho Chung

**Affiliations:** Research School of Biology, Australian National University, Canberra, ACT 0200, Australia; E-Mails: anna.robinson@anu.edu.au (A.R.); shin-ho.chung@anu.edu.au (S.-H.C.)

**Keywords:** hanatoxin, Kv2.1, voltage sensor, gating modifier toxin, molecular dynamics

## Abstract

Hanatoxin 1 (HaTx1) is a polypeptide toxin isolated from spider venoms. HaTx1 inhibits the voltage-gated potassium channel kv2.1 potently with nanomolar affinities. Its receptor site has been shown to contain the S3b-S4a paddle of the voltage sensor (VS). Here, the binding of HaTx1 to the VSs of human Kv2.1 in the open and resting states are examined using a molecular docking method and molecular dynamics. Molecular docking calculations predict two distinct binding modes for the VS in the resting state. In the two binding modes, the toxin binds the S3b-S4a from S2 and S3 helices, or from S1 and S4 helices. Both modes are found to be stable when embedded in a lipid bilayer. Only the mode in which the toxin binds the S3b-S4a paddle from S2 and S3 helices is consistent with mutagenesis experiments, and considered to be correct. The toxin is then docked to the VS in the open state, and the toxin-VS interactions are found to be less favorable. Computational mutagenesis calculations performed on F278R and E281K mutant VSs show that the mutations may reduce toxin binding affinity by weakening the non-bonded interactions between the toxin and the VS. Overall, our calculations reproduce a wide range of experimental data, and suggest that HaTx1 binds to the S3b-S4a paddle of Kv2.1 from S2 and S3 helices.

## 1. Introduction

Many gating modifier toxins isolated from animal venoms interfere with the gating mechanisms of biological ion channels such as voltage-gated potassium (Kv) channels. For example, various spider toxins are believed to partition into the membrane [1,2,3,4], bind to the voltage sensor (VS) of the channel, which is formed by S1–S4 helices, and interfere with channel gating [5]. Conformational changes of the VS that are required for opening the channel are presumably hindered by the toxin. The toxins may bind to and stabilize the channel in the resting state, such that the channel becomes harder to open [6]. In the presence of the toxin, the channel can still open but a stronger depolarization is required, thus shifting the voltage-activity curve to the right [5]. 

Hanatoxin 1 (HaTx1) [7], *Scodra griseipes* toxin 1 (SGTx1) [8], the voltage sensor toxin 1 (VSTx1) [9], heteropodatoxin 2 (HpTx2) [10] and phrixotoxin (PaTx) [11], all isolated from spider venoms, are some of the most well characterized gating-modifier toxins to date [5]. All these toxins are polypeptides consisting of 25–40 amino acids. HaTx1 inhibits both Kv2.1 and Kv4.2 channels effectively at a concentration of 500 nM [7], but does not inhibit the archeabacterial Kv channel KvAP [12], whereas VSTx1 selectively binds KvAP but not Kv2.1 [12]. In contrast, HpTx2 and PaTx are selective inhibitors of Kv4 channels [11,13]. The toxin backbones are interconnected by three disulfide bonds forming the inhibitor cysteine knot motif [14]. The solution structures of these toxins suggest that a hydrophobic patch primarily formed by phenylalanine, methionine and tryptophan residues is conserved, although the size and shape of this patch varies [2,15,16]. This hydrophobic patch, together with the polar segment encompassing it, is believed to be critical for toxin binding [3,5,17]. The detailed process by which one of the gating-modifier toxins penetrates into the membrane has been examined using molecular dynamics (MD) simulations [18,19,20,21]. These showed that the hydrophobic patch of the toxin interacts with the core of bilayer, while its polar region retains attractive bonds with lipid head groups and water molecules. 

Since the discovery of HaTx1 in 1995 [7], the modes of interactions between the VS and several gating modifier toxins have been studied extensively using mutagenesis techniques [5,6,22,23,24,25]. Experiments performed on Kv2.1 have identified a number of important residues, primarily located on the S3 helix of the VS, that are likely involved in the binding of HaTx1 [23,24,25]. In particular, the substitution of the glutamate residue at position 277 of rat Kv2.1 with lysine or tyrosine, or the phenylalanine residue at position 274 with arginine or glycine, causes a large reduction in the affinity of HaTx1 [24]. These two residues are located on S3b, Glu277 near the polar heads of the lipid bilayer facing the extracellular space and Phe274 further below, near the hydrophobic core of the membrane. HaTx1 thus appears to penetrate partially into the membrane, with its hydrophobic patch interacting with the binding groove near S3. The hydrophobic residues from the toxin and the VS form hydrophobic clusters, while the basic residues from the toxin make hydrogen bonds and salt bridges with acidic residues from the VS. While Kv2.1 binds gating modifiers primarily through the S3 helix, the prokaryotic Kv channel KvAP may bind VSTx1 primarily through the S4 helix [12]. This suggests that VSTx1 and HaTx1 bind to different regions of the VS domain. 

**Figure 1:**
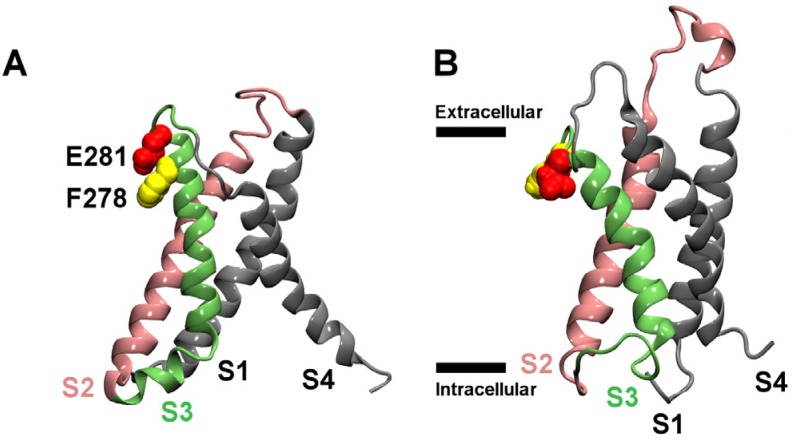
Homology models of the voltage sensors (VSs) of Kv2.1 in the resting (**A**) and open (**B**) states. The side chains of two key residues in the periplasmic segment of the S3 helix, Phe278 and Glu281, are highlighted. In (**B**), the boundary of the membrane is indicated with horizontal bars.

Here we examine the binding of HaTx1 to the VSs of Kv2.1 in the open (VS_O_) and resting (VS_R_) states, using a molecular docking method and MD simulations. The model structures of the VS in the resting and open states are displayed in Figure 1. The positions of two helices, S3 and S4, are noticeably different between the resting state and open state conformations. In the open state, the S3 helix is moved inward, whereas the S4 helix is moved further outward. We use molecular docking to predict possible binding modes between the toxin and the VS_R_, which provides the best receptor site for the toxin. Each of the distinct binding modes predicted are subsequently equilibrated in a lipid bilayer and a box of explicit water for 50 ns, and the mode consistent with experiment is considered to be correct. The toxin is then docked to the VS_O_ assuming an orientation similar to that in the correct binding mode of HaTx1-VS_R_. The calculations suggest that HaTx1 bind to its primary receptor site, the S3b-S4a paddle [24], from S2 and S3 helices.

## 2. Results and Discussion

### 2.1. Binding to the VS_R_

First we examine the binding of HaTx1 to the isolated VS of Kv2.1 in the resting state. The isolated VS rather than the full channel with the pore domain attached is used for computational efficiency, because the pore domain is not required for toxin binding [26] and isolated VS domains are stable when incorporated into lipid bilayers [27]. 

**Figure 2:**
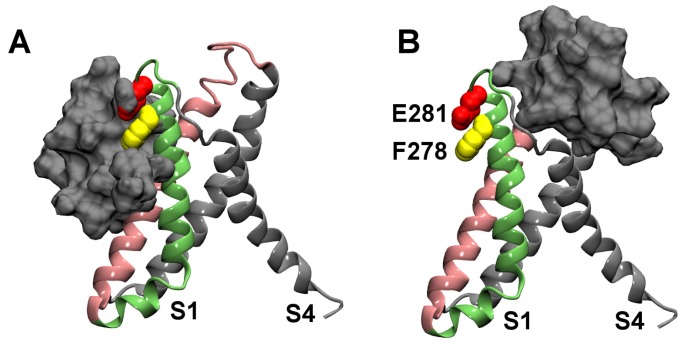
The two distinct binding modes of HaTx1 and the resting state VS of Kv2.1 predicted from molecular docking calculations. Molecular surface of HaTx1 is shown in silver.

Two distinct binding modes, as displayed in Figure 2, are predicted by molecular docking calculations. In the first binding mode, HaTx1 binds to the periplasmic half of the VS_R_ from S2 and S3 helices (Figure 2A), whereas in the second binding mode, the toxin binds to the VS_R_ from S1 and S4 helices (Figure 2B). These two binding modes represent the only two orientations by which HaTx1 can bind the extracellular segment of VS_R_. The two key residues Phe278 and Glu281, corresponding to Phe274 and Glu281 in rat Kv2.1, are in close proximity to the toxin in the first binding mode, but they are far away from the toxin in the second binding mode. In both binding modes, the functional surface of HaTx1 consists of six hydrophobic residues, Leu5, Phe6, Phe23, Trp30, Phe32 and Phe34, corresponding to the key residues of the closely related toxin SGTx1 determined experimentally [17]. The second binding mode is consistent with that observed for VSTx1 and KvAP in the multiscale simulations of Wee *et al*. [21]. However, HaTx1 and VSTx1 may bind to different regions of the VS [12]. The first binding mode is more consistent with mutagenesis data, and therefore is more likely to be the correct binding mode. Subsequent MD simulations performed on the two binding modes support this proposal.

Since the molecular docking calculations are performed in the absence of membrane, water and ions, it is important to equilibrate the structures predicted in a more realistic environment. The two structures of HaTx1-VS_R_ are embedded in a lipid bilayer and a box of explicit water and ions, and subsequently simulated for 50 ns each using MD without restraints, as described in the Methods section. 

The initial complexes determined from docking, when embedded in the lipid bilayer, reveal that the toxin is partially buried in the hydrophobic core of the bilayer. In the first binding mode, the center of mass (COM) of HaTx1, located 6 Å above the center of the bilayer at the start of the simulation, shifts upward by about 6 Å over the simulation period of 50 ns. About half of the toxin remains buried in the lipids at the end of the simulation. In the second binding mode, the distance between the COM of HaTx1 and the bilayer center along the bilayer normal (18–19 Å) does not change significantly during the equilibration. The overall architecture of the VS_R_ is rigid in both binding modes, with the maximum RMSD of the backbone atoms of the VS_R_ with respect to the starting structure being less than 3 Å. 

**Figure 3:**
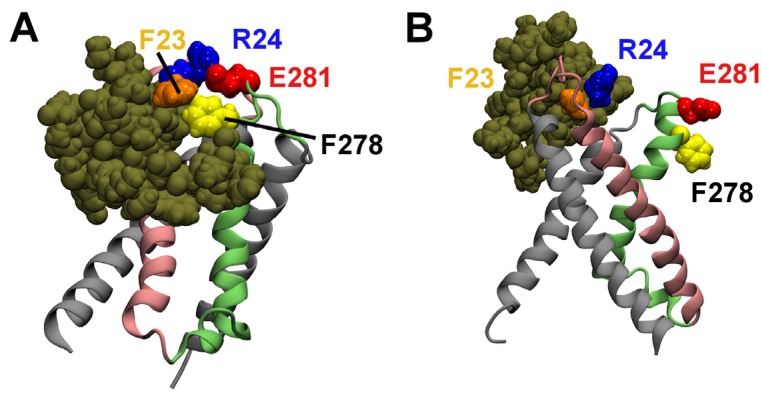
HaTx1 bound to the resting state VS of Kv2.1 after 50 ns of molecular dynamics (MD) simulation. The two possible binding modes are shown. Toxin molecule is in tan. The side chains of two toxin residues, Phe23 and Arg24, and two VS residues, Phe278 and Glu281, are highlighted.

The positions of HaTx1 relative to the VS_R_ of Kv2.1 after the equilibration are shown in Figure 3. Water molecules, ions and lipids are not shown in the figure. In the first binding mode, the VS residue Glu281 is observed to form a salt bridge with the toxin residue Arg24 (Figure 3A). Here, a salt bridge is considered to be formed if the distance is ≤ 4 Å between a side-chain oxygen of an acidic residue and a side-chain nitrogen of a basic residue [28]. In addition, the VS residue Phe278 forms hydrophobic interactions with the phenylalanine residue at position 23 of HaTx1. The distance between HaTx1 Trp30 and the center of the bilayer fluctuates around an average of 6 Å over the last 10 ns. This is in reasonable agreement with experimental measurements which suggest that Trp30 is about 8.5 Å from the bilayer center on the binding of HaTx1 to membranes [6]. In the second binding mode, the toxin does not move toward Phe278 and Glu281 of the VS during the simulation period of 50 ns, and no salt bridge between the VS and toxin is observed. Therefore, the first binding mode is more consistent with mutagenesis experiments, and is considered to be correct.

### 2.2. Binding to the VS_O_

Experimentally it has been shown that HaTx1 binds to the VS of Kv2.1 in both open and resting states, but the binding to the open state is less energetically favorable [6]. If the binding mode of HaTx1-VS_R_ shown in Figure 3A were correct, less favorable toxin-VS interactions should be formed if the open conformation of the VS were used in the docking. Following this hypothesis, we perform docking calculations of HaTx1 with the VS_O_ of Kv2.1. The complex in which the position of HaTx1 relative to the VS is similar to that in Figure 3A is selected for subsequent MD simulations. 

**Figure 4:**
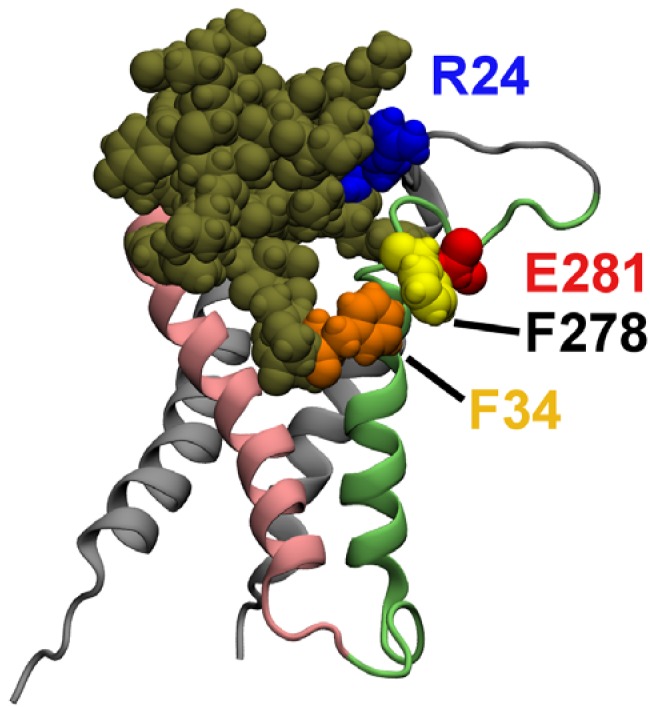
HaTx1 bound to the open state VS of Kv2.1 after 50 ns of MD simulation. Two toxin residues, Phe34 and Arg24, and two VS residues, Phe278 and Glu281, are highlighted.

Figure 4 displays the structure of HaTx1 bound to the VS_O_ after 50 ns of simulation. The VS residue Glu281 does not form a salt bridge with the toxin residue Arg24, although the VS residue Phe278 interacts favorably with the toxin residue Phe34. The residue Trp30 of HaTx1 is about 9.5 Å from the center of the bilayer, comparable to the value of 8.5 Å determined experimentally [6]. Without a salt bridge, the interactions in the toxin-VS_O _complex are less favorable than that in the toxin-VS_R_ complex. This would predict that HaTx1 binds more strongly to the VS_R_, which is consistent with experiment [6].

### 2.3. Computational Mutagenesis

If the model of HaTx1-VS_R_ shown in Figure 3A were representative of the binding of HaTx1 to Kv2.1 in real systems, the model should be able to reproduce the effect of single mutation to the VS on toxin binding observed experimentally. The F278R and E281K mutations have been shown experimentally to cause the largest effect on the binding affinity of HaTx1 [24]. Therefore, we perform two single mutations, F278R and E281K, to the HaTx1-VS_R_ complex shown in Figure 3A. The mutant complexes are subsequently simulated for 20 ns without restraints.

**Figure 5:**
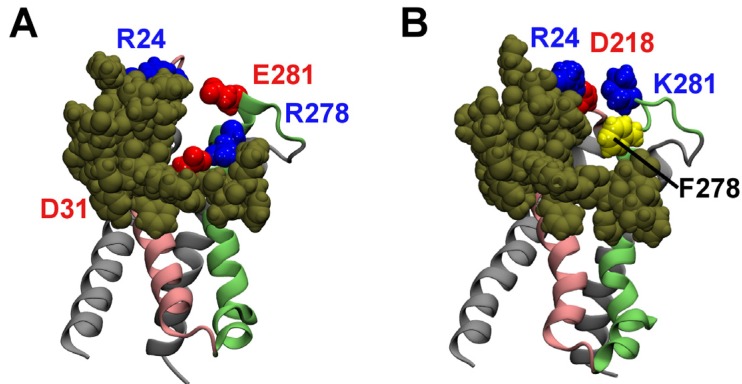
HaTx1 bound to the F278R (**A**) and E281K (**B**) mutant VSs of Kv2.1 in the resting state after 20 ns of simulation.

The complex of HaTx1-F278R VS_R_ after 20 ns of simulation is shown in Figure 5A. The position of HaTx1 relative to the F278R VS_R_ is similar to that observed before the mutation. In this complex, the salt bridge Arg24–Glu281 is broken. However, a new salt bridge, Asp31–Arg278 is formed. The complex of HaTx1-E281K VS_R_ after 20 ns of simulation is shown in Figure 5B. Again, HaTx1 remains bound to the mutant VS after the equilibration. Similar to the HaTx1-F278R VS_R _complex, the salt bridge Arg24–Glu281 is broken but an equivalent salt bridge (Arg24–Asp218) is formed in the complex HaTx1-E281K VS_R_. Thus, the overall strength of electrostatic interactions between the toxin and the VS_R_ appears to be similar between the wild type and mutant VSs. 

To quantify the strength of non-polar interactions between the toxin and the wild type and mutant VSs, we calculate the solvent accessible surface area (SASA) of the interface between the toxin and the VS. SASA is widely used to estimate the non-polar component of solvation free energies in the empirical methods Molecular Mechanic/Poisson-Boltzmann Surface Area (MM/PBSA) and Molecular Mechanic/Generalized Born Surface Area (MM/GBSA) for estimating free energies of ligand binding [29,30]. Results tabulated in Table 1 show that SASA of HaTx1-VS_R_ is 790 Å^2^, which is significantly higher than the values of 690 Å^2^ for HaTx1-F278R VS_R_ and 750 Å^2^ for HaTx1-E281K VS_R_. The SASA values are monotonically related to the corresponding free energies of binding inferred from experimental data (Table 1), with a linear correlation coefficient of −0.9. However, such a correlation does not demonstrate that the toxin binds the mutant VSs less strongly, because SASA only approximates one component of the free energy of binding. Other components such as entropy, internal energies of the toxin and VS, VS-lipid interactions, toxin-lipid interactions and polar non-bonded interactions between the toxin-VS, may not cancel out between the wild type and mutant VSs. We have attempted to derive the potential of mean force profile for the toxin binding and calculate the dissociation constant *K*_d_, but found that the convergence of the profile is poor within the time scale accessible to us. Nevertheless, calculations of SASA indicate that non-polar interactions between the toxin and the VS are weakened by the mutations. 

It is possible that HaTx1 binds to the wild type and mutant VSs of Kv2.1 equally strongly, but the ability of HaTx1 to inhibit the movement of the VS is significantly reduced due to the different interactions formed between the toxin and mutant VSs. Such uncoupling of binding from activity has already been demonstrated in the binding of scorpion β-toxin Css4 to sodium channels [31]. As shown in the binding modes of HaTx1 to the mutant VSs displayed in Figure 5, both mutations interrupt the salt bridge formed by Glu281 of the voltage sensor. We therefore cannot rule out the possibility that the F278R and E281K mutations disrupt toxin activity by interrupting the Arg24–Glu281 salt bridge formed in the HaTx1-wild type VS complex. Future binding assay experiments should help clarify this issue. 

**Table 1:** The solvent accessible surface area (SASA) of the binding interface between HaTx1 and the wild type (WT) and mutant VSs of Kv2.1 in the resting state.

VS	SASA (Å^2^)	Δ*G*_bind_ (kT)
WT	790 ± 20	−16.1
F278R	690 ± 27	−9.7
E281K	751 ± 53	−11.0

Δ*G*_bind_ is calculated as Δ*G*_bind_ = *kT* ln(*K*_d_/*C*_0_), where *C*_0_ is 1 M. The dissociation constant *K*_d_ values are 103 nM, 61.6 μM and 17.3 μM for WT, F278R and E281K mutants, respectively [24].

### 2.4. Membrane Partition

**Figure 6:**
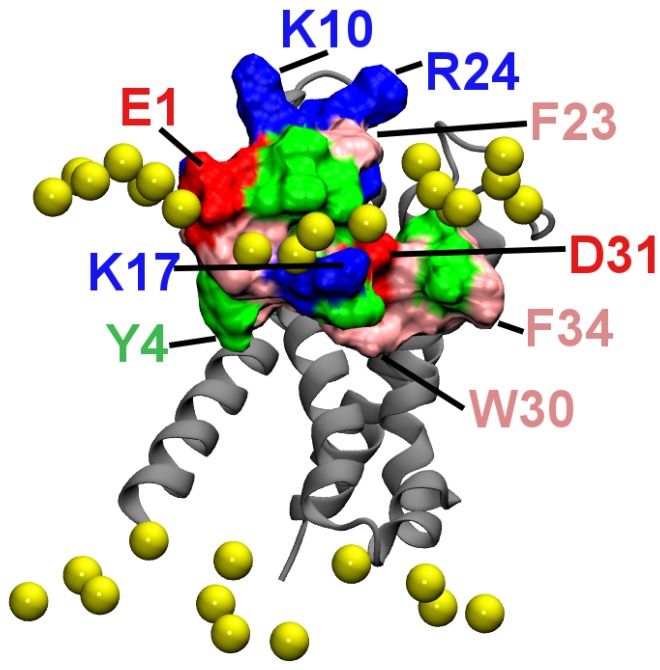
The position of HaTx1 relative to lipids in the simulation corresponding to that of Figure 3A. The phosphorus atoms of lipids near the toxin are shown as yellow spheres, and the backbone of the VS as silver ribbons.

Figure 6 shows the position of HaTx1 relative to lipid head groups in the simulation corresponding to that in Figure 3A. It is seen that several hydrophobic residues, including Tyr4, Trp30 and Phe34 penetrate into the hydrophobic core of the bilayer, whereas the charged residues Glu1, Lys17 and Asp31 are located in the polar head group region. By forming favorable hydrophobic interactions with lipid tails and hydrogen bonds with lipid head groups, the toxin-VS_R_ complex is stabilized in the bilayer. About half of the toxin is buried in the lipids and the other half in the water phase. The membrane partition of HaTx1 observed here is consistent with that observed experimentally for various similar gating modifier toxins such as VSTx1 [1], SGTx1 [3], HaTx1 [6], and GsMTx4 [32]. In the bound complex, the hydrophobic patch of HaTx1 consisting of residues Leu5, Phe6, Phe23, Trp30, Phe32 and Phe34 is interacting primarily with the VS and not the lipids. This patch is nearly parallel to the bilayer normal. However, it should be perpendicular to the bilayer normal and parallel to the bilayer plane on the binding of HaTx1 to pure lipid bilayers according to previous computational studies of closely related toxins [19,20]. Therefore, a membrane-bound HaTx1 would need to reorient on binding to the VS. 

In the previous computational studies of scorpion α- and β-toxins [33,34], which are gating modifiers of voltage gated sodium (Nav) channels, both scorpion α- and β-toxins do not partition into membranes on binding. The differences in the membrane partition abilities of HaTx1 and scorpion toxins may be related to the distinct function of these toxins. Scorpion β-toxins shift the voltage-activity curve of Nav channels to the left, such that the channel requires less depolarized potentials to open [35]. In contrast, HaTx1 shifts the voltage-activity curve of Kv2.1 to the right, such that the channel opening requires stronger depolarization [7]. It is known that the VS_R_ especially the S4 helix is more buried in the membrane compared to the VS_O_ [36,37]. Thus, membrane partitioning may be required for HaTx1, because the toxin has to bind the resting state VS within the membrane.

## 3. Methods

### 3.1. Initial Structures

HaTx1 binds the most potently to the VS in the resting state [6]. Homology models are generated for the VS of Kv2.1 in the open and resting states, using the automated homology modeling server SWISS-MODEL [38,39,40]. The sequence of *Homo sapiens* Kv2.1 is obtained from the NCBI protein database (NCBI entry NP_004966.1). The model of Kv1.2 in the resting state [41] and the crystal structure of Kv1.2 in the open state (PDB ID: 3LUT) [42,43] are used as templates. The sequence identity between the VSs of Kv1.2 and Kv2.1 is ~40%, above 30% that is required for reliable homology models to be generated [44]. The solution structure 1D1H [15] is used for HaTx1.

### 3.2. Molecular Docking

We use the rigid-body molecular docking program ZDOCK 3.0.1 [45] to survey possible binding modes of HaTx1 and the VSs of Kv2.1. Each docking calculation generates 500 structures. The structures in which the toxin is located in the cytoplasmic half of the VS are eliminated, because HaTx1 binds to the periplasmic segment of the VS according to experiment [6]. Subsequently cluster analysis with a toxin backbone RMSD cutoff of 20 Å is performed on the remaining structures to identify distinct binding modes by HaTx1.

### 3.3. Molecular Dynamics Simulations

The bound complexes predicted by molecular docking are embedded in a POPC (2-oleoyl-1-palmitoyl-*sn*-glycero-3-phosphocholine) bilayer solvated with a rectangular box of explicit water and 0.2 M KCl. The simulation boxes, 70–80 Å in each dimension, contained approximately 160 lipids, 32 K^+^/Cl^−^ ions and 8500 water molecules. Each system is then equilibrated for 50 ns without restraints. 

All MD simulations are performed using NAMD 2.8 [46], with periodic boundary conditions and a 2-fs time step. The CHARMM27/CMAP force field for proteins [47,48], C36 force field for lipids [49] and the TIP3P model for water [50] are used. The switch and cutoff distances for short-range interactions are 8.0 Å and 12.0 Å, respectively. The particle mesh Ewald method is used to describe long-range electrostatic interactions, with a maximum grid spacing of 1.0 Å. The SHAKE [51] and SETTLE [52] algorithms are used to keep the bond lengths in the system rigid. The short-range nonbonded interactions and the long-range electrostatic forces are computed every one and two steps, respectively. The Langevin dynamics and the Nosé-Hoover Langevin Piston method [53] are used for maintaining a temperature of 300 K and a pressure of 1 bar on average in the system. Trajectories are saved every 20 ps for analysis. Molecular graphics are generated using VMD [54].

## 4. Conclusions

In this work using a combination of molecular docking methods and molecular dynamics simulations, models of HaTx1 bound to the VS of human Kv2.1 in the open and resting states are constructed. The models reproduce a wide range of experimental observations, including the relative affinity of HaTx1 for the VS in the open and resting states, and the reduction in toxin affinity due to mutations to two key residues (Phe278 and Glu281) of the VS. It is found that HaTx1 binds to its primary receptor site S3b-S4a paddle from S2 and S3 helices.
